# The Roles and Activities of Medical Simulation Technicians in Poland: A Preliminary Exploratory Study

**DOI:** 10.3390/healthcare14070944

**Published:** 2026-04-03

**Authors:** Jakub Zalewski, Mateusz Ptak, Dawid Obłój, Joanna Grzesik Gąsior, Katarzyna Zalewska

**Affiliations:** 1 State University of Applied Sciences in Krosno, Rynek 1, 38-400 Krosno, Poland; jakub.zalewski@pans.krosno.pl (J.Z.); joanna.grzesik-gasior@pans.krosno.pl (J.G.G.); 2 Institute of Health Sciences, University of Opole, ul. Katowicka 68, 45-061 Opole, Poland; mateusz.ptak@uni.opole.pl (M.P.); dawid.obloj@uni.opole.pl (D.O.)

**Keywords:** simulation technicians, roles, education, activities

## Abstract

**Introduction**: The role of medical simulation technicians varies across countries and institutions. Technicians ensure simulators function according to the designed training scenarios and manage their physiological responses, such as breathing and circulation. They are responsible for ongoing maintenance, repairs, and software installation or updates. Additionally, they manage audio–video systems, including recording simulation sessions, organizing live broadcasts, and preparing materials for post-training review and analysis. These responsibilities suggest that technicians contribute to the technical and organizational foundations of simulation-based education. **Methods**: This exploratory study used a predominantly quantitative survey design with an additional qualitative component. An open-ended question allowed respondents to freely express their opinions and reflections regarding the professional responsibilities of medical simulation technicians. This mixed-methods approach enabled both statistical analysis and deeper insight into the technicians’ perspectives. **Results**: Thirty-five simulation technicians participated in the study. Most respondents were men (71.43%), while women accounted for 28.57%. Respondents reported performing a broad range of technical, organizational, and selected educational support tasks. **Conclusions**: This exploratory study suggests that medical simulation technicians in Poland perform a broad range of technical, organizational, and selected educational support tasks. The findings also indicate local variability in role configuration and unclear boundaries between technical and educational responsibilities. Given the limited sample size and purposive recruitment strategy, the results should be interpreted cautiously.

## 1. Introduction

Medical simulation has become an integral element of contemporary healthcare education, responding to increasing demands for quality of care and patient safety [1,2,3]. The effective implementation of simulation-based teaching requires the involvement of a team of professionals with diverse competencies, not only educators or clinicians but also individuals responsible for the technical and organizational aspects of the educational process. The role of the medical simulation technician is gaining growing importance as an important member of the Medical Simulation Center (MSC) team [4]. Across simulation centers worldwide, technicians support the technical and organizational conditions required for simulation-based training, thereby contributing to the development of clinical skills and interpersonal competencies among healthcare professionals. Thanks to their work, future doctors, nurses, and other medical professionals can learn in environments that closely replicate real-life conditions [5,6].

Simulation teams around the world define the role of the technician in various ways. The medical simulation technician is not only expected to have a sound understanding of the equipment from a technical perspective but also familiarity with educational methodology and the implementation of simulation-based training [7]. Their responsibilities include, among other duties, the preparation and operation of simulation equipment, ranging from basic task trainers used for fundamental skills acquisition to advanced patient simulators. Technicians ensure that simulators operate in line with the designated training scenario and control their physiological responses, such as breathing or circulation. They also conduct ongoing maintenance and repairs and install or update software systems. Additionally, they are responsible for managing audio–video systems, which include recording simulation sessions, organizing live broadcasts, and preparing materials for post-training review and analysis [8,9]. Simulation technicians also manage inventory and the organization of training spaces, ensuring the availability of disposable equipment and the proper setup of simulation rooms. They support both instructors and learners by guiding them in the operation of simulators and task trainers. Their tasks also involve documenting simulation activities and analyzing outcomes, allowing for the optimization of training processes and the improvement of educational quality [10].

In many countries, the role of the medical simulation technician—also referred to as simulation technologist or simulation operations specialist—has been clearly defined and standardized. Such professionals are expected to have knowledge in areas such as internal medicine, surgery, obstetrics, or emergency medicine. They contribute to medical education by helping to ensure a realistic representation of clinical situations. Their work requires not only technical and analytical skills but also communication abilities, flexibility, and the ability to operate in a dynamic educational environment. As a result, students and educators can improve not only practical professional skills but also competencies in communication and time-pressure work—within a safe, controlled setting [7]. In the United States, Canada, and Western Europe, simulation technicians often hold specialized certifications such as the Certified Healthcare Simulation Operations Specialist (CHSOS), awarded after passing an exam administered by the Society for Simulation in Healthcare (SSH) [11]. The INACSL Standards of Best Practice highlight the technician’s role in preparing and conducting simulation sessions, managing equipment, and ensuring an important level of realism in educational activities. In the United Kingdom, organizations such as NHS Health Education England and the Association for Simulated Practice in Healthcare (ASPiH) define the technician’s role as encompassing operational, technical, and educational support. They also describe the “simulation technician” as an integral member of the simulation team and promote the standardization of career paths and competencies within national health workforce development strategies [12,13].

In contrast to many countries in Western and Northern Europe, where professional certification for simulation technicians (e.g., CHSOS from SSH) is more widespread, in Poland, such formal credentials are only beginning to gain traction. More training programs and professional development courses focused on simulator operation and scenario management are emerging. Many institutions draw on the experience of international organizations such as SESAM (Society in Europe for Simulation Applied to Medicine).

Despite the growing importance of this profession, the role of the medical simulation technician in Poland has not yet been formally defined. In practice, it is shaped locally within academic institutions, which may lead to variations in assigned tasks and expected qualifications. Because this role combines technical, organizational, and selected educational support functions, it remains important to better describe its scope in the Polish context.

Therefore, the aim of this study was to identify and describe the range of tasks performed by medical simulation technicians in Poland, considering practices reported in academic centers. Given the exploratory nature of the study, the findings are intended to provide preliminary descriptive insight rather than a basis for broad generalization.

## 2. Materials and Methods

### 2.1. Study Design

This was an exploratory cross-sectional survey study with a quantitative design and an additional qualitative component. The qualitative element consisted of one open-ended question that allowed respondents to freely express their opinions and reflections regarding the responsibilities of a medical simulation technician. This question was intended to capture perspectives that may not have been fully reflected in the predefined list of tasks. Therefore, the study was designed as an exploratory mixed-methods study aimed both at identifying the frequency of specific tasks performed by simulation technicians and at exploring how they conceptualize the scope and boundaries of their professional role.

### 2.2. Questionnaire

A study-specific questionnaire was developed by a team of experts in healthcare simulation, particularly those familiar with the work of simulation technicians. It consisted of items enabling the sociodemographic characterization of the study group and items related to task scope, divided into the following categories:

- Technical and IT tasks (4 items);
- Technical and organizational tasks (2 items);
- Educational, organizational, and representational tasks (6 items);
- Administrative and clerical tasks (5 items);
- Technical and moulage-related tasks (3 items);
- Technical and maintenance tasks (2 items);
- One open-ended question aimed at gathering respondents’ opinions on the appropriate scope of duties for medical simulation technicians, including the opportunity to suggest additional tasks (if not covered by the survey) and to indicate activities that should not be part of this professional role.

All closed-ended items were rated using a 5-point Likert scale, where

1—Never;
2—Rarely;
3—Sometimes;
4—Often;
5—Very often.

In total, the questionnaire comprised 23 questions. Before data collection, the draft questionnaire was reviewed by researchers experienced in healthcare simulation to assess the clarity and relevance of the items. Data were collected using Google Forms.

The questionnaire was developed as an exploratory descriptive tool intended to map the range of duties performed by medical simulation technicians and the frequency with which those duties were reported. The grouped task sections were introduced to organize questionnaire content and structure the presentation of findings; they were not intended as psychometric subscales.

### 2.3. Participants and Recruitment

The data analyzed in the present manuscript were derived from the initial exploratory stage of a broader ongoing study. At this stage, the questionnaire was distributed to approximately 15–16 higher education institutions in Poland, roughly one institution per voivodeship. A total of 35 complete questionnaires were included in the analysis.

Potential respondents were identified and invited to participate in the study using a contact list created by the research team. For this purpose, email invitations were sent to selected universities, requesting participation in the study. This approach enabled outreach to simulation technicians working at several types of higher education institutions that host MSCs within their institutional structures. The inclusion criterion was active involvement in tasks related to the technical and operational support of medical simulation activities in an MSC, regardless of the respondent’s formal job title. Individuals not performing simulation-related technical duties or not working in a setting using medical simulation were excluded from participation. Because no official national registry of medical simulation technicians exists in Poland, and because simulation-related duties are sometimes performed by staff employed under different formal job titles (e.g., technical, administrative, or IT positions), the sampling frame should be considered purposive and exploratory rather than exhaustive.

### 2.4. Ethics

Before completing the questionnaire, each participant provided informed and voluntary consent to participate in the study. They were also informed about the study’s purpose.

The study was approved by the Research Ethics Committee of the University of Opole, under reference no. 68/2022. The study was reported in accordance with the STROBE checklist guidelines to ensure the highest possible quality and transparency of the manuscript.

### 2.5. Data Analysis

Quantitative data were summarized using descriptive statistics. Categorical variables are presented as frequencies and percentages, whereas continuous variables are presented as means and standard deviations. For each Likert-scale item, mean (M) and standard deviation (SD) values were additionally calculated to support item-level interpretation of the frequency with which specific duties were performed. No aggregate domain scores were calculated. The grouped task sections served an organizational purpose only and were used to structure questionnaire content and the presentation of findings. Because the items within each section represented distinct professional activities rather than indicators of a single latent construct, the sections were not treated as psychometric subscales.

Responses to the open-ended question were subjected to a systematic thematic content analysis, which proceeded in the following steps: (1) two researchers independently reviewed and initially coded all responses; (2) codes were compared and differences were discussed until a consensus was reached that ensured consistency; (3) recurring codes were subsequently grouped into broader categories reflecting participants’ perceptions of the scope, boundaries, and expectations associated with the role of a medical simulation technician.

To ensure transparency, the results of the qualitative analysis are presented in a structured format, i.e., according to the developed categories. This presentation allows qualitative data to provide meaningful analytical insights rather than merely serving as illustrative examples and complements the quantitative analysis by offering a deeper understanding of how medical simulation technicians perceive their professional responsibilities.

## 3. Results

### 3.1. Participant Characteristics

Table 1 presents the sociodemographic and institutional characteristics of the respondents, including age, sex, education, the type of institution in which the MSC operates, and the educational programs supported by the center. A total of 35 medical simulation technicians participated in the study. Most respondents were men (71.43%), while women accounted for 28.57% of the sample. The mean age was 38.70 years (SD = 8.86). Most respondents held a higher education degree (82.85%). All respondents worked in MSCs operating within universities or higher education institutions. The educational programs most supported by the represented centers included nursing, medicine, midwifery, and emergency medical services.

### 3.2. Task Frequency Ratings

To complement the frequency distributions shown in the figures, Table 2 presents item-level mean and standard deviation values for all task-frequency items.

### 3.3. Technical and IT Tasks

Analysis of the collected data showed that most respondents (62.86%) reported very frequent involvement in operating audiovisual components of simulation sessions (camera operation, recording, sound, etc.). An even larger proportion (74.29%) declared very frequent engagement in managing simulator software during simulations. Furthermore, 85.71% of the respondents indicated that they very frequently performed basic technical preparations for training sessions—i.e., setting up simulators, manikins, task trainers, and other simulation-related devices.

**Figure 1:**
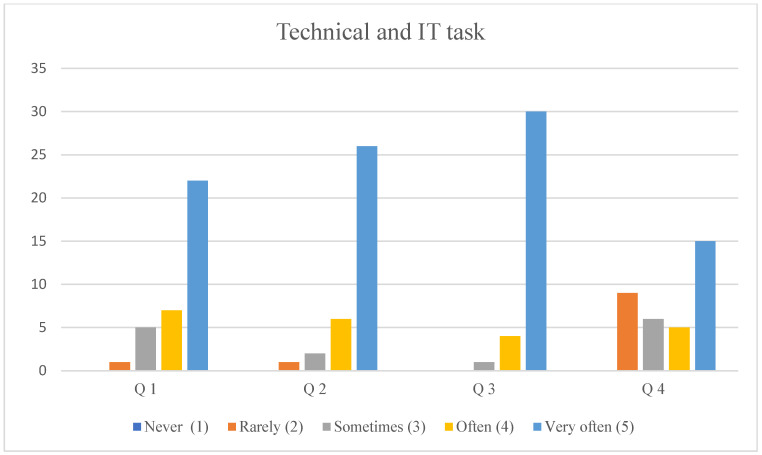
Frequency of performing technical and IT tasks according to the Likert scale. Legends: Q 1—operating audiovisual systems during simulation sessions (camera operation, recording, sound, etc.); Q 2—operating simulator software during simulation sessions; Q 3—technical preparation of sessions, i.e., basic activities related to the preparation of simulators, manikins, trainers, and other devices related to teaching in Medical Simulation Centers; Q 4—programming simulation scenarios/patient conditions in dedicated software integrated with the simulator.

Programming simulation scenarios or patient conditions using software integrated with simulators was not performed by 25.73% of the respondents, while 17.14% stated they did it rarely or sometimes. On the other hand, 42.86% reported performing this task very frequently (see Figure 1).

### 3.4. Technical and Organizational Tasks

Regarding technical and organizational activities, responses were relatively consistent. Specifically, 77.14% of the respondents reported that they very frequently prepare and provide single-use medical equipment for simulation sessions, while 80.00% indicated that they very frequently perform tasks related to maintaining the overall infrastructure of the MSC.

### 3.5. Educational, Organizational, and Representational Tasks

Educational, organizational, and representational tasks showed greater variability in responses. In the question concerning participation in simulation scenarios in roles such as a standardized patient, a patient’s family member, or additional medical personnel, 42.86% of the respondents indicated that they perform such tasks occasionally. A similar distribution of responses regarding the frequency of performing a given activity was observed for Question 11, which referred to assisting with or conducting educational sessions for external groups. These activities included simulations, courses, instructional sessions, or lectures organized for individuals outside the student community, including representatives of various professional groups as well as visitors to the MSC (also from abroad).

**Figure 2:**
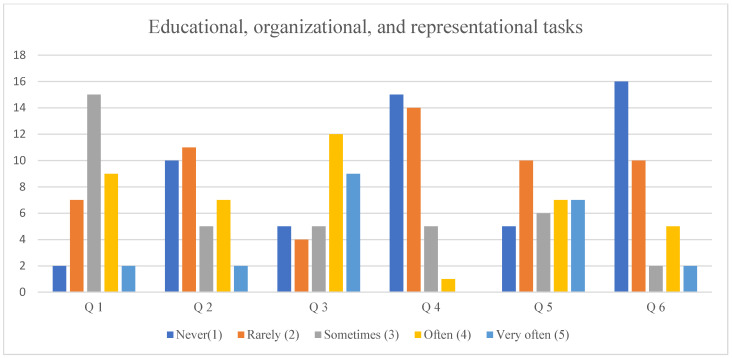
Educational, organizational, and representational tasks according to the Likert scale. Legends: Q 1—participation in a simulation scenario as a standardized patient, patient’s family, additional medical staff, etc.; Q 2—preparation of a simulation scenario or lesson plan at the Medical Simulation Center; Q 3—conducting pre-briefing and training sessions for students on the functionality of simulation equipment in a given room; Q 4—conducting/participating in debriefings; Q 5—assisting with or conducting activities for external groups, including simulations, courses, training sessions, or lectures for non-students, professional groups, or visitors to the Simulation Center; Q 6—involvement in scientific research on the work and functioning of Medical Simulation Centers.

Furthermore, 45.71% of the respondents reported that they had never been involved in scientific research related to the work and functioning of MSC. The distribution of responses is shown in Figure 2.

### 3.6. Administrative and Clerical Tasks

The analysis of responses in this category showed a noticeable variation in task frequency. As many as 60.00% of the respondents declared that they very frequently performed inventory management of disposable medical supplies and simulation devices, including maintaining records of equipment in both teaching rooms and storage areas. This highlights resource management as one of the most regularly fulfilled responsibilities.

Conversely, 48.57% of respondents indicated that they never managed the Simulation Center’s website or social media accounts (e.g., Facebook, Twitter), nor did they engage in public relations activities related to the unit.

### 3.7. Technical and Moulage-Related Tasks

Within this category, particular attention was drawn to the task of recovering disposable medical supplies. 48.57% of the respondents stated that they performed this task very frequently, making it one of the most common in this area. This task involves not only technical aspects but also logistics and resource optimization within simulation centers.

### 3.8. Technical and Maintenance Tasks

In the question concerning off-site equipment maintenance and repair—defined as reporting malfunctions and coordinating with service technicians or equipment manufacturers—68.57% of the respondents stated that they performed these tasks at least occasionally. A similar pattern was observed in Question 22 (with 60.00% of respondents reporting at least occasional involvement), which asked about independently performing minor repairs, modifications, and maintenance of software and simulation devices. These findings indicate a consistent level of involvement in the ongoing maintenance of technical infrastructure within simulation centers.

### 3.9. Free-Form Comments

Thematic analysis of responses to the open-ended question identified three main themes: (1) perceived scope and boundaries of the technician role, (2) collaboration within the educational team, and (3) context-dependent expectations regarding additional technical responsibilities. Overall, respondents emphasized the need for a clearer role definition, particularly about the distinction between technical support and educational responsibility.

(1) Perceived role scope of a simulation technician

Respondents described the technician role primarily in terms of maintaining simulation infrastructure, organizing teaching spaces, and ensuring the availability and functionality of equipment and consumables. At the same time, several participants emphasized that activities such as writing lesson plans, designing teaching processes, or leading debriefings should remain primarily within the responsibility of teaching staff, even if technicians provide important technical support. As one respondent stated:

*“Writing scenarios or planning lessons should not fall under the technician’s responsibilities. Yes, the technician should be consulted, as they know the equipment and its availability, but this is not their responsibility.”*

A similar view was expressed regarding the technician’s participation in debriefing sessions:

*“Participating in or especially leading debriefings falls under educational competencies. A technician may support this process but not conduct it.”*

(2) Collaboration within an educational team

The second theme concerned collaboration within the educational team. Respondents emphasized that effective simulation-based education requires close cooperation between technicians and faculty members. Technicians were described as important contributors during the planning and organizational stages, particularly because of their knowledge of equipment, room availability, and the status of consumable supplies. One respondent noted:

*“However, it is advisable to consult with them during the planning phase. Technicians are familiar with the equipment and its capabilities, know the status of disposable supplies, and are aware of the availability of individual rooms at the Center.”*

(3) Context-dependent expectations regarding additional responsibilities

The third theme reflected the view that the technician’s role may vary depending on the organizational structure, staffing model, and resources of a given center. Respondents suggested that some duties are shaped locally and may differ between institutions, provided that the overall division of responsibilities remains clear. As one participant stated:

*“The technician’s role will differ depending on the organizational and staffing structure of a given center. What is important is that the role is defined in a way that allows the center to function smoothly, and the team to feel satisfaction in their work and learning.”*

These insights underscore the necessity of clearly defining the professional competencies of medical simulation technicians and incorporating their perspectives into the organization and delivery of simulation-based education, not as lecturers, but as specialized team members responsible for the technical and organizational foundation of the training environment.

## 4. Discussion

The present study provides preliminary insight into the role of medical simulation technicians in Poland and the range of responsibilities they perform in Medical Simulation Centers. The findings indicate that technicians are involved not only in core technical tasks, such as operating simulators and audiovisual systems or preparing equipment for sessions, but also in selected organizational and educational support activities. This suggests that their role extends beyond narrow technical maintenance and includes functions that contribute to the continuity and effectiveness of simulation-based education.

As highlighted in a systematic review conducted by Tellefson et al. (2025) [14], simulation technicians play a multifaceted role within the educational team, supporting the educational process and ensuring the technological functionality of the infrastructure. The authors emphasize the need to clarify their competencies and formalize collaboration protocols with academic staff. The present findings reflect the specific nature of simulation technicians’ work in the Polish context and add to the international discussion on the development of this role.

Similar conclusions were presented by Bailey et al. (2015) [4], who stressed that the technician’s role encompasses not only technical elements but also involvement in scenario preparation, coordination of simulation sessions, and educational support. According to the authors, clearly defining the responsibilities of simulation technicians is essential for developing career paths and increasing the profession’s prestige.

Moreover, the present study highlights the variation in the scope of duties performed by medical simulation technicians across institutions. Respondents’ answers suggest that the assigned tasks differ significantly depending on local staffing resources, organizational structures, and the chosen operational model of the MSC. This observation is consistent with the literature indicating a lack of unified competency frameworks, particularly in countries where the profession is still evolving (Roche et al., 2022) [15].

The qualitative findings further suggest that one of the central issues is the unclear boundary between technical and educational responsibilities. Respondents often described technicians as important contributors to the planning and operational delivery of simulation sessions, while simultaneously indicating that lesson planning, leading debriefings, or taking primary responsibility for educational content should remain within the domain of teaching staff. This finding is conceptually important because it points to a role that is neither exclusively technical nor fully educational.

This apparent ambivalence may reflect the hybrid nature of the simulation technician role. On the one hand, technicians contribute directly to the design, delivery, and continuity of simulation-based learning. On the other hand, they may still perceive their professional identity as distinct from that of faculty members or instructors. Rather than representing a contradiction, this pattern may indicate that simulation technicians occupy an intermediate position between technical support and educational participation.

This position is also supported by research conducted by Hughes et al. (2021) [16], which concluded that technicians may function as so-called “silent partners in education”—present and involved, yet not responsible for delivering substantive content.

The study also pointed to the wide spectrum of organizational and representational tasks performed by technicians, such as AV system operation, equipment testing, inventory management, and participation in promotional events. The smooth functioning of a simulation center would be significantly hindered without their technical support, which underlines the need to define this role and provide technicians with real opportunities for professional development, e.g., through training, certification (CHSOS, SSH), or integration into teaching teams.

Additionally, the results of this study indicate that both the broad scope of tasks and the lack of clearly defined competency boundaries may contribute to ambiguity in the distribution of responsibilities that exceed the technician’s formal role. Participants frequently pointed out the absence of a clear division between technical and educational duties, emphasizing the need to define distinct roles within simulation center teams. On the one hand, they reported being actively involved in lesson preparation, scenario management, and technical equipment operation; on the other, they expressed reluctance to take on educational tasks such as debriefing or scenario creation.

These reflections are consistent with international research. For example, Roche et al. (2022) [15] stress the importance of defining the technician’s competencies in relation to their operational role, rather than their educational function, while also recognizing their crucial contribution to the effectiveness of simulation-based education. Bailey et al. (2015) [4] reach similar conclusions, emphasizing that a clear job description is essential for the continued professionalization of the role.

Respondents also highlighted that simulation technicians must be capable not only of technical efficiency but also of effective response to unexpected situations—such as equipment failures, disruptions in scenarios, or organizational issues. Their responses often indicated a need for developing operational competencies under dynamic and high-stress conditions. These observations align with international practices, including the “Bug Busters” program described by Crawford et al. (2019) [17], which trains technicians using simulated failures and crisis scenarios. This example illustrates one possible approach to supporting the development of operational competencies in this professional group.

Taken together, the findings suggest that medical simulation technicians in Poland perform a broad and locally variable range of tasks and that the boundaries of this role are not always clearly defined. Given the exploratory design and limited sample size, these conclusions should be interpreted cautiously. However, the study provides preliminary evidence that may support further discussion on clearer role descriptions, professional development pathways, and future multicenter research on simulation workforce organization in Poland.

## 5. Limitations

An important limitation of this study relates to the recruitment strategy. The analyzed data come from the initial exploratory stage of a broader ongoing study and include a relatively small purposive sample of 35 respondents recruited from approximately 15–16 higher education institutions in Poland. Because no official national registry of medical simulation technicians exists, and because simulation-related duties may be performed under heterogeneous formal job titles (e.g., technical, administrative, or IT positions), it was not possible to establish a complete and verifiable sampling frame. Therefore, the findings cannot be considered nationally representative.

The questionnaire included only limited contextual information about respondents’ institutions. As a result, we were unable to characterize participating centers in terms of size, funding model, infrastructure, staffing structure, or formal role configuration of simulation technicians. Therefore, the results should be interpreted with caution, considering their exploratory nature and the fact that they reflect the perspective of only a portion of medical simulation technicians in Poland.

Another limitation is that the questionnaire was a study-specific exploratory instrument and was not formally validated psychometrically. Importantly, the grouped task sections served an organizational rather than a psychometric function. Because the items within each section represented distinct professional activities rather than indicators of a single latent construct, no domain-level composite indices were calculated. Accordingly, the findings should be interpreted as preliminary descriptive evidence rather than as results derived from a validated measurement tool.

Despite these limitations, the study provides preliminary exploratory insight into the scope of duties performed by medical simulation technicians in Poland and may serve as a basis for further multicenter research in this area.

## 6. Conclusions

This exploratory study indicates that medical simulation technicians in Poland perform a broad range of technical, organizational, and selected educational support tasks. The findings also suggest that the scope of this role varies across institutions and that the boundaries between technical and educational responsibilities are not always clearly defined. Given the limited sample size and purposive recruitment strategy, the results should be interpreted cautiously. Nevertheless, they provide preliminary evidence that may inform future multicenter research and further discussion on clearer role descriptions and professional development pathways for medical simulation technicians in Poland.

## Figures and Tables

**Table 1:** Sociodemographic and institutional characteristics of respondents (N = 35).

Characteristic		N	%
Age (years)	M = 38.70, SD = 8.86		
Sex	Female	10	28.57
	Male	25	71.43
Education	Secondary	6	17.15
	Higher	29	82.85
Educational programs supported by the MSC *	Medicine	10	19.60
	Nursing	20	39.22
	Midwifery	9	17.64
	Paramedic/Emergency Medical Services	10	19.62
	Physiotherapy	2	3.92

M: mean, SD: standard deviation. * Multiple responses were possible.

**Table 2:** Item-level descriptive statistics for task frequency ratings.

Task Domain	Task Item	Mean (SD)
Technical and IT tasks	Operating audiovisual systems during simulation sessions (e.g., cameras, recording, sound).	4.40 (0.83)
	Operating simulator software during simulation sessions.	4.63 (0.72)
	Technical preparation of sessions, including basic preparation of simulators, manikins, trainers, and other equipment used in Medical Smulation Centers.	4.83 (0.45)
	Programming simulation scenarios and patient conditions using dedicated software integrated with the simulator.	3.69 (1.24)
Technical and organizational tasks	Preparing and stocking disposable medical supplies for sessions, including transporting equipment between rooms and replenishing shortages in cabinets, countertops, and resuscitation carts.	4.74 (0.50)
	Taking care of the overall infrastructure of the Simulation Center.	4.74 (0.55)
Educational, organizational, and representational tasks	Participating in simulation scenarios as a standardized patient, patient’s family member, an additional healthcare professional, or a similar role.	3.06 (0.95)
	Preparing a simulation scenario or a session plan for activities conducted in the Medical Simulation Center.	2.40 (1.26)
	Conducting pre-briefing and instruction for students regarding the functionality of simulation equipment in each room.	3.54 (1.29)
	Conducting or participating in debriefing sessions.	1.80 (0.78)
	Assisting with or conducting activities for external groups, including simulations, courses, training sessions, or lectures for non-students, professional groups, or visitors to the Simulation Center (including international visitors).	3.09 (1.36)
	Involvement in scientific research related to the work and functioning of Medical Simulation Centers.	2.09 (1.29)
Administrative and clerical tasks	Inventorying disposable medical supplies and simulation equipment (equipment records) in training rooms and storage areas.	4.17 (1.10)
	Handling procurement procedures, preparing purchase requests, and purchasing equipment for the Simulation Center, including disposable medical supplies, software, simulators, trainers, manikins, and other educational devices.	3.83 (1.32)
	Supervising and documenting the disposal of medical waste.	2.80 (1.48)
	Performing administrative tasks.	3.46 (1.27)
	Managing the website and social media of the Simulation Center (e.g., Facebook, Twitter) and/or performing other tasks related to the public image of the unit.	2.43 (1.53)
Technical and moulage-related tasks	Performing full moulage or visual preparation of simulators, manikins, standardized patients, and related elements for simulation sessions.	3.74 (1.10)
	Creating simulation devices, trainers, or medical equipment for teaching purposes using available or recycled materials, where possible.	2.74 (1.25)
	Recovering disposable medical supplies for further educational use (e.g., resealing opened packages, repurposing used materials, and assembling new equipment sets).	4.06 (1.26)
Technical and maintenance tasks	Off-site repair and maintenance of equipment, including reporting failures and cooperating with technical staff and service personnel representing the software/equipment manufacturer.	4.51 (0.84)
	On-site repair and maintenance of equipment, including minor in-house work aimed at repairing, modifying, or maintaining software, equipment, and devices used in simulation.	4.29 (1.08)

Note. Items were rated on a 5-point Likert scale, where 1 = never, 2 = rarely, 3 = sometimes, 4 = often, and 5 = very often. SD = standard deviation.

## Data Availability

The data supporting the findings of this study are not publicly available because they include qualitative responses from a relatively small professional group, which may increase the risk of indirect identification. An anonymized minimal dataset supporting the main findings can be made available to the editorial office for evaluation and to qualified researchers upon reasonable request to the corresponding author.

## Abbreviations

The following abbreviations are used in this manuscript:

MSC	Medical Simulation Center
CHSOS	Certified Healthcare Simulation Operations Specialist
SSH	Society for Simulation in Healthcare
ASPiH	Association for Simulated Practice in Healthcare
SESAM	Society in Europe for Simulation Applied to Medicine
AV	Audio–Video
IT	Information Technology
